# Multilesional pyoderma gangrenosum is associated with longer healing times and higher rates of underlying comorbidities: A single-center prospective registry study

**DOI:** 10.1016/j.jdin.2023.05.009

**Published:** 2023-05-27

**Authors:** Hannah Zhao, Arash Mostaghimi, Jonathan M. Sisley, Alex G. Ortega-Loayza

**Affiliations:** a School of Medicine, Oregon Health & Science University, Portland, Oregon; b Department of Dermatology, Brigham & Women’s Hospital, Boston, Massachusetts; c Department of Dermatology, Oregon Health & Science University, Portland, Oregon

**Keywords:** inflammation/inflammatory, medical dermatology, neutrophils/neutrophilic, pyoderma gangrenosum, ulcers, wounds and wound healing

*To the Editor:* Pyoderma gangrenosum (PG) is a rare neutrophilic dermatosis with variable presentations.^1^ In this single-center prospective PG registry study, we evaluate the clinical differences between patients presenting with single versus multilesional PG.

Patients evaluated between December 2019 and June 2022 were identified from the Pyoderma Gangrenosum Study Registry (PYGAS) at Oregon Health and Science University.^2^ All patients had a PARACELSUS score of at least 10. Clinical, demographic, and treatment data was extracted for further analysis. PG-related past medical history and clinical characteristics were based on baseline visits. Patients were classified into single versus multilesional PG groups based on number of lesions developed throughout their treatment course and subsequent visits. The mean follow-up time between visits was 44.0 days (SD+/45.5). Two-sample *t*-tests and Chi-squared tests were used to compare the 2 groups, with statistical significance established as *P* ≤ .05.

We identified 95 patients with classic (ulcerative), vegetative, and bullous PG, of which 39 (41.1%) presented with a single ulcer and 56 (58.9%) presented with 2 or more ulcers. The mean age of presentation for single and multilesional PG was 52.6 (SD ± 17.7) and 55.0 (SD ± 13.9) years respectively (Table I). Overall, the location of PG lesions was similar between both groups. Ulcers were the most common in the lower extremity; they comprised 84.6% of the single ulcer group and were the location of at least 1 ulcer for 85.7% of the multi ulcer group. Ulcers located in the upper extremity were only found in the multi ulcer group, and the only case of head and neck PG was found in the single ulcer group (Fig 1).

**Table I:** Single vs multi-ulcer patient demographics, clinical characteristics, and healing outcomes

	Single ulcer	Multi-ulcer	*P* value
Age, y, mean (SD)	52.6 (17.7)	55.0 (13.9)	.481
Sex (number of patients, %)			
Male	13 (33.3)	12 (21.4)	.841
Female	26 (66.7)	44 (78.6)	.031
Type of PG (number of patients, %)			
Classic (ulcerative)	39 (100)	51 (91.1)	.206
Vegetative	0	4 (9.27)	.046
Bullous	0	1 (1.82)	.317
Location of ulcers (number of patients, %)			
Lower extremities	33 (84.6)	48 (85.7)	.096
Upper extremities	0	1 (1.78)	.317
Trunk (including breast)	4 (10.3)	6 (10.7)	.527
Head/neck	1 (2.56)	0	.317
Genitals	1 (2.56)	1 (1.78)	1.000
Wound characteristics			
Healing time (months) (SD)	5.52 (4.26)	7.80 (4.62)	.017
Wounds that have not healed (number of patients, %)	15 (28.3)	38 (17.7)	.001
Wound age (months) (SD)	11.3 (8.30)	9.80 (3.50)	.230
Wound size (cm^2^) (SD)	49.8 (13.5)	51.4 (12.9)	.561
Comorbidities (number of patients, %)			
Hx of inflammatory rheumatological disease	2 (20.0)	8 (80.0)	.058
Hx of solid cancer	6 (40.0)	9 (60.0)	.439
Hx of hematological disease	2 (66.7)	1 (33.3)	.564
Hx of hidradenitis suppuritiva	1 (25.0)	3 (75.0)	.317
Hx of DVT	2 (14.3)	12 (85.7)	.008
Hx of PE	1 (33.3)	3 (66.7)	.317
Hx of IBD (Crohn’s, UC, unknown)	3 (23.0)	10 (76.9)	.052
Hx of hematological malignancy	1 (50.0)	1 (50.0)	1.000

*DVT*, Deep vein thrombosis; *Hx*, history; *IBD*, inflammatory bowel disease; *PE*, pulmonary embolism; *UC*, ulcerative colitis.

**Fig 1:**
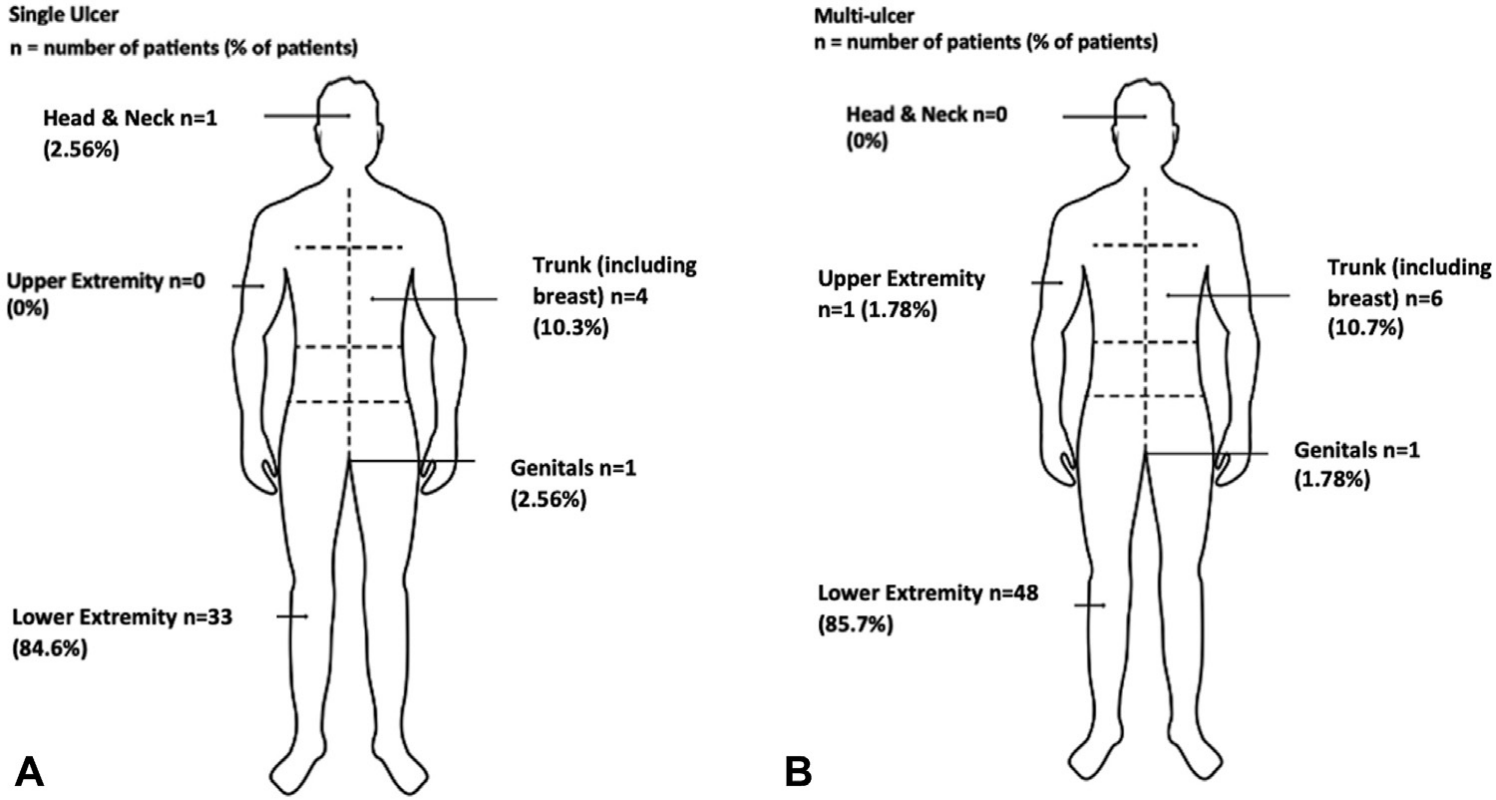
**A** and **B,** Single ulcer and multi ulcer distributions. This includes the number of patients with ulcers located in the 5 body distributions along with the respective percentages.

The target lesion (the largest ulcer on bidimensional plane) mean wound size (cm^2^) for single and multilesional groups were 49.8 (SD ± 13.5) and 51.4 (SD ± 12.9) respectively. Wound age at the time of initial baseline presentation in clinic was 11.3 months and 9.8 months for single and multilesional patients respectively. The mean healing time was 5.5 months for the single ulcer group and 7.8 months for the multilesional group (*P* = .017). Of 53 patients with nonhealed wounds at the time of analysis, 38 (71.7%) were found in the multilesional group and only 15 (28.3%) were found in the single ulcer group (*P* = .001) (Table I).

Evaluation of comorbidities demonstrated higher rates of inflammatory diseases and thromboembolism in the multilesional patients compared to single ulcer patients (Table I). A history of DVT was associated with statistically significant results (2 cases vs 12 cases, *P* = .008), which might be explained by the interplay of inflammation and thrombosis (Table I).^3^

There was not an adequate sample size of healed patients to conduct a multivariate analysis. As this is an initial descriptive study, future studies will include a multivariate assessment to rule out confounding factors modulating healing time and comorbidities.

In conclusion, this study described differences in wound healing time and comorbidities between patients with single versus multiple lesions of pyoderma. These findings build on prior data suggesting that ulcer size at initial presentation is the most important predictor of healing time, and that ulcer number influences disease severity.^4^^,^^5^

## Conflicts of interest

Dr Alex Ortega-Loayza has served in advisory boards for Janssen, BMS, and Boehringer Ingelheim, and as a consultant for Genentech and Guidepoint. He has also received research grants from Eli Lilly Company, OHSU School of Medicine Gerlinger research award, and Medical Research Foundation of Oregon. Dr Mostaghimi reports no conflicts of interest relevant to this manuscript. He has received consulting fees from Pfizer, hims, Digital Diagnostics, Concert, Lilly, Abbvie, Equillium, Boehringer, Ingelheim, LEO, ACOM, and Digital Diagnostics.
